# Viral co-infection with SARS-CoV-2 in hospitalized Peruvian patients during the years 2020-2021

**DOI:** 10.17843/rpmesp.2026.431.15162

**Published:** 2026-03-27

**Authors:** Carlos Augusto Yabar, Karolyn Vega-Chozo, Eduardo Miranda-Ulloa, Flor de María Peceros-Pelaez, Manuel Terrazas-Aranibar, Adolfo Marcelo, María Paquita García, María Elena López, Rubén Vásquez, Katia Zúñiga, Haydee Alvarado, Renso López-Liñán, Heriberto Arévalo, Nancy Rojas-Serrano

**Affiliations:** 1 Innovation and Diagnostic Support Unit, National Institute of Health, Lima, Peru.; 2 Faculty of Human Medicine, Universidad de San Martín de Porres, Lima, Peru.; 3 Faculty of Natural and Mathematical Sciences, Universidad Nacional Federico Villarreal, Lima, Peru.; 4 National Reference Laboratory for Sexually Transmitted Viruses, National Institute of Health, Lima, Peru.; 5 National Reference Laboratory for Vector-borne and Viral Zoonotic Diseases, National Institute of Health, Lima, Peru.; 6 María Auxiliadora Hospital, Lima, Peru.; 7 Regional Hospital of Loreto, Loreto, Peru.; 8 Universidad Nacional de la Amazonia Peruana, Loreto, Peru.; 9 Regional Public Health Reference Laboratory of San Martín, San Martín, Peru.; 10 National Reference Laboratory for Vaccine-Preventable Diseases, National Institute of Health, Lima, Peru.

**Keywords:** Coinfection, SARS-CoV-2 Infection, Severe Acute Respiratory Syndrome, Laboratory Diagnosis

## Abstract

The study aimed to identify coinfections of SARS-CoV-2 with other viruses in patients with moderate and severe COVID-19. 98 patients from Lima, San Martín, and Loreto were selected, from whom nasopharyngeal secretion, blood, and urine samples were taken and analyzed using serological and molecular tests to detect HTLV, HIV, hepatitis B, dengue, Chikungunya, Zika, and a panel of respiratory viruses (influenza A/B, rhinovirus, respiratory syncytial virus, metapneumovirus, and adenovirus). The results showed two cases of coinfection (2/98; 2%): one with SARS-CoV-2/rhinovirus and another with SARS-CoV-2/HTLV-2. Both patients presented with COVID-19-associated pneumonia but progressed favorably. It is concluded that the frequency of viral coinfection in patients from health facilities in the Peruvian coast and jungle with moderate and severe cases of COVID-19 was low.

## INTRODUCTION

Its first report, COVID-19 continues to manifest worldwide, with more than 778 million confirmed cases and 7.1 million deaths by the end of 2025 ^1^. During 2020 and 2021, 28.5% of the deaths recorded worldwide were concentrated in South America ^2^, with Peru being the country with the highest per capita mortality rate for COVID-19 in the world ^3^.

While the severity of COVID-19 has been associated with metabolic, genetic, and demographic factors ^4^^-^^6^, it has recently been demonstrated that co-infections with other respiratory viruses also contribute to greater disease severity ^7^^,^^8^. In fact, it has been reported that the fatality rate in patients co-infected with more than one virus was three times higher compared to those who presented with a single infection ^8^. This evidence suggests that viral co-infections can modify the clinical course of the disease and constitute indicators of poor prognosis.

It should be noted that during the SARS-CoV-2 pandemic, Peru also faced various local viral epidemics, including dengue (DENV) ^9^, Chikungunya virus (CHIKV) ^10^, hepatitis B virus (HBV), and human immunodeficiency virus type 1 (HIV-1) ^11^, human T-cell lymphotropic virus (HTLV) ^12^, as well as seasonal respiratory viruses ^13^. The sustained circulation of these viral agents could have generated favorable conditions for the occurrence of co-infections with SARS-CoV-2. In this context, with the aim of determining the frequency of viral co-infections, a multicenter study was proposed in areas where other endemic viruses have also been reported, including subjects with moderate and severe clinical manifestations of COVID-19.

**KEY MESSAGES**

Motivation for the study. During the SARS-CoV-2 pandemic, the Peruvian population was exposed to different local viral agents, increasing the possibility of acquiring co-infections and, consequently, the risk of developing severe COVID-19.

Main findings. Two cases of viral co-infection with rhinovirus and HTLV-2 were reported. The latter is the first case reported in the world.

Implications. This study provides information on the presence of viral infectious agents that occur simultaneously in a pandemic context and under territorial characteristics, which is relevant from a clinical and epidemiological perspective.

## THE STUDY

A cross-sectional study was conducted to recruit patients with a confirmed diagnosis of SARS-CoV-2 via reverse transcription polymerase chain reaction (qRT-PCR) between January 26 and August 13, 2021. Inclusion criteria were age ≥18 years, both sexes, and hospitalization with moderate or severe COVID-19 according to MINSA's Epidemiological Alert AE-016-2020. Patients with more than two comorbidities (diabetes, obesity, hypertension, cancer) were excluded.

Patients were recruited by convenience from four public hospitals: Hospital María Auxiliadora and Hospital San Juan de Lurigancho (Lima), Hospital II-2 of Tarapoto (San Martín), and Hospital Felipe Arriola Iglesias (Loreto). The selection of centers was made by convenience following a national call.

Samples included nasopharyngeal secretion (for molecular tests), blood (5 mL in tubes without anticoagulant), and urine (10-20 mL in sterile containers). All were transported in a cold chain (0-10 °C) to the National Institute of Health in Lima for processing.

Data collection was carried out using COVID-19 clinical-epidemiological forms approved by MINSA and the CIEI-INS.

For the molecular diagnosis of respiratory viruses, RNA was extracted from nasal and pharyngeal swabs using the QIAamp® DSP Viral RNA kit. Multiplex qRT-PCR with the TaqMan system was applied in three panels: Panel 1 (Influenza A, Influenza B, GAPDH), Panel 2 (respiratory syncytial virus, metapneumovirus, rhinovirus), and Panel 3 (adenovirus). Amplifications were performed in a RotorGene Q thermal cycler.

Detection of dengue (DENV), Chikungunya (CHIKV), and Zika (ZIKV) was carried out through viral RNA extraction from sera using an automated magnetic bead system and subsequent qRT-PCR with the Logix Smart™ ZDC kit. For ZIKV in urine, the Hollycon® AE2130 system and qRT-PCR with primers targeting the E gene were used, using a BIORAD CFX96 thermal cycler.

Serological tests included the detection of antigens and antibodies against HIV, HTLV-1/2, hepatitis B, and arboviruses. For HBV, HBsAg ELISA (WANTAI) was used; for HTLV, the HTLV 1/2 Immunoblot test (Fujirebio); and for HIV, the Murex kit with confirmation via INNO-LIA™ HIV I/II Score. Serological diagnosis of dengue was performed with IgM ELISA (TARIKI), while for Chikungunya, the EUROIMMUN Chik IgM indirect immunofluorescence test was used.

This design allowed for the evaluation of viral co-infections in patients hospitalized with moderate and severe COVID-19 in different regions of Peru, under strict ethical and methodological criteria.

The study received ethical approval from the CIEI-INS (code OC-022-20). Participation was voluntary, with informed consent signed by patients or relatives, guaranteeing principles of medical ethics and scientific research.

## FINDINGS

During the recruitment period, 98 patients with a confirmed diagnosis of SARS-CoV-2 and moderate or severe clinical status were included. The majority were treated in Lima (52%), although it was not determined if they came from other regions. Males predominated (58.2%), and the average age was 52 years. Regarding clinical presentation, 43.8% of the cases presented with severe clinical symptoms. Comorbidities were identified in 33% of cases, primarily diabetes (13%). Only 20.4% presented an unfavorable prognosis. The majority received treatment with enoxaparin and dexamethasone (table 1).

**Table 1:** Characteristics of patients with COVID-19.

Characteristics		HMA Lima n=27 (%)	HSJL n=24 (%)	Hospital II-2 Tarapoto (San Martín) n=18 (%)	Felipe Arriola Iglesias Hospital (Loreto) n=29 (%)	Total n=98 (%)
Mean age (years)		59	48	52	49	52
Sex						
	Male	17 (63.0)	15 (62.5)	8 (44.4)	17 (58.6)	57 (58.2)
	Female	10 (37.0)	9 (37.5)	10 (55.6)	12 (41.4)	41 (41.8)
Mechanical ventilation						
	Yes	0 (0.0)	1 (4.2)	2 (11.1)	1 (3.4)	4 (4.1)
	No	13 (48.1)	23 (95.8)	16 (88.9)	28 (96.6)	80 (81.6)
	ND	14 (51.9)	0 (0.0)	0 (0.0)	0 (0.0)	14 (14.3)
Prognosis						
	Stable	11 (40.7)	5 (20.8)	9 (50.0)	6 (20.7)	31 (31.6)
	Unfavorable	5 (18.5)	4 (16.7)	4 (22.2)	7 (24.1)	20 (20.4)
	Favorable	9 (33.3)	13 (54.2)	5 (27.8)	14 (48.3)	41 (41.8)
	ND	2 (7.4)	2 (8.3)	0 (0.0)	2 (6.9)	6 (6.1)

HMA: María Auxiliadora Hospital; HSJL: San Juan de Lurigancho Hospital; ND: Not determined

Regarding viral co-infection, two cases were detected (2%) (table 2). The first corresponded to a 46-year-old man in Lima with SARS-CoV-2/Rhinovirus co-infection, a moderate case of pneumonia, and no comorbidities. He was hospitalized for seven days and treated with ceftriaxona, clindamycin, and other drugs, progressing favorably. The second case was a 57-year-old woman in Iquitos with SARS-CoV-2/HTLV-2 co-infection, who presented with cough, dyspnea, and hyporexia. Her vital signs showed blood pressure 110/80, heart rate 107, respiratory rate 26, and saturation 93%. After seven days of symptoms, she received ceftriaxona, enoxaparin, and omeprazole, with a positive evolution.

**Table 2:** Clinical and laboratory characteristics in the two cases of viral coinfection.

Characteristics	First case	Second case
Coinfection	Rhinovirus	HTLV-2
Age (years)	46	57
Clinical classification	Moderate	Severe
Comorbidity	None	Obesity and hypertension
Mechanical ventilation	No	No
Outcome	Favorable	Favorable
Pharmacological therapy	Ceftriaxone, Clindamycin, Ketoprofen, Tramadol, Ranitidine	Ceftriaxone, enoxaparin, azithromycin, dexamethasone, metamizole and omeprazole
UREA (mg/dL)	ND	34
Creatinine (mg/dL)	ND	0.61
Glucose (mg/dL)	ND	127.2
AST (Ul/L)	ND	69
LDH (Ul/L)	ND	950
ALT (Ul/L)	ND	95
C-reactive protein (mg/L)	ND	134.8

ND: Not determined; AST (GOT): Aspartate aminotransferase; LDH: Lactate dehydrogenase; ALT (GPT): Alanine aminotransferase.

## DISCUSSION

In this study, we conducted a search for cases of viral co-infection in 98 hospitalized patients positive for SARS-CoV-2 through a study carried out in a city on the coast and two in the jungle of Peru. Our findings revealed the presence of two cases of viral co-infection (2%), whose frequency is within the range identified by other studies (0.6 - 20%) ^7^^-^^9^^,^^12^^,^^15^^-^^18^. However, the wide difference in the frequency of viral co-infection may be due to the number and type of biological samples, population characteristics, study design, the presence of endemic viral infections, and even the diagnostic methods used.

In the case of García-Vidal *et al.*^14^, they found a viral co-infection prevalence of 0.6%, similar to our findings. However, this could be related to the type of population selected, as, compared to that study, our report only included hospitalized patients, in whom bacterial co-infection could also occur as previously demonstrated ^15^. Furthermore, it is likely that the detection of viral agents is subject to the limitations of the methodology used, such as in the case of real-time PCR, whose sensitivity depends on the sequence matching capacity of the primers and probes in the viral RNA.

Regarding the cases identified, the detection of co-infection between SARS-CoV-2 and respiratory viruses, mainly rhinovirus, is relatively frequent ^16^. Although rhinovirus infection does not cause major symptoms in adults, the case of co-infection with SARS-CoV-2 exhibited a severe manifestation, which is interesting because the patient showed no comorbidities. In this regard, Le Glass *et al.*^16^ demonstrated that patients with SARS-CoV-2 and rhinovirus co-infection manifested severe clinical forms, with a higher probability of entering intensive care and dying. These data reveal that SARS-CoV-2/rhinovirus co-infection could trigger severe symptoms in adult patients. Given that the case shown in our study did not present any type of comorbidity and considering previous evidence, it is possible that a co-infection between SARS-CoV-2 and rhinovirus was the cause of the severe clinical manifestation in the patient; however, this requires further studies.

Regarding the second case, there are previous reports of co-infection between SARS-CoV-2 and HTLV ^12^^,^^17^^,^^18^, of which Avila Dextre *et al*. ^12^ reported a possible co-infection between the pandemic virus and HTLV-2. Accordingly, out of a total of 22 serologically confirmed carriers of HTLV and SARS-CoV-2, seven samples were typed as HTLV-2. However, the confirmatory test they used to diagnose the co-infection was based on antibody detection, so it was not possible to know with certainty the patient's HTLV infection status at the time of contracting SARS-CoV-2, mainly for two reasons: 1) Since 2000, more than 200 families from the Shipibo-Konibo native communities of the jungle have lived in Lima ^19^, a coastal city where HTLV-2 has never been reported. This suggests that it is not possible to know if some healthy individuals in these communities were infected with HTLV-2 before or after being infected with SARS-CoV-2 or if some subjects infected with HTLV-2 were transmitting to others simultaneously. 2) The researchers mentioned that some positive cases of SARS-CoV-2 were confirmed by IgM/IgG serological tests (ECLIA assay); however, these patients were previously vaccinated against SARS-CoV-2, which increases the possibility of false-positive results. All these arguments support the fact that our study would be the first confirmed report of co-infection between SARS-CoV-2 and HTLV-2 not only in Peru but also in the world. However, we also do not have the certainty that it was an acute active co-infection with SARS-CoV-2.

Analyzing the clinical characteristics of this particular case, it is important to note that the patient co-infected with SARS-CoV-2/HTLV-2 presented a severe clinical form of COVID-19 with a favorable evolution; the patient presented obesity and hypertension, factors that aggravate COVID-19 and therefore could have played an important role in the clinical manifestations. In the report by Avila Dextre *et al.*^12^, seven subjects presented moderate clinical manifestations; however, none were associated with COVID-19 severity. In this regard, recall bias could be interfering in the interpretation of these results since it was observed that the clinical information collected was based on the participant's memory. Therefore, the evidence is insufficient to determine if the presence of HTLV-2 in a patient with SARS-CoV-2 could trigger moderate or severe clinical forms of COVID-19.

It is important to note that the present study has some limitations. First, the sample size was small, mainly due to the difficulty of accessing patients in critical condition, the refusal of relatives, and entry restrictions to the Intensive Care Unit during the pandemic. Second, the heterogeneous distribution of viral agents at the national level may have limited the detection of viruses responsible for local epidemics such as DENV, CHIKV, ZIKV, HIV, and HBV, whose clinical manifestations can be confused with or exacerbate COVID-19. Third, the clinical-epidemiological information of the selected subjects was incomplete due to restricted access to clinical documentation, which was recorded by the healthcare personnel of the health facilities as part of routine care, without them being part of the research team.

In conclusion, a low frequency of viral co-infection was found in Peruvian patients with moderate and severe forms of COVID-19 in some health facilities on the Peruvian coast and jungle. These findings contribute additional data for the design of future epidemiological, clinical, and laboratory surveillance strategies to identify cases of viral co-infection. Likewise, it opens the possibility of new lines of research in the context of co-infections and the development of multicenter studies that strengthen the understanding and response to these public health challenges.
