# Supplementary material for: Viral co-infection with SARS-CoV-2 in hospitalized Peruvian patients during the years 2020-2021
Source: Rev Peru Med Exp Salud Publica. 2026 Mar 27;43(1):104–9. doi: 10.17843/rpmesp.2026.431.15162 (PMC13245989; doi:10.17843/rpmesp.2026.431.15162)
Supplement: Translation

# Translation: Coinfección viral con SARS-CoV-2 en pacientes peruanos hospitalizados durante los años 2020-2021

**Authors:** Carlos Augusto Yabar, Karolyn Vega-Chozo, Eduardo Miranda-Ulloa, Flor de María Peceros-Pelaez, Manuel Terrazas-Aranibar, Adolfo Marcelo, María Paquita García, María Elena López, Rubén Vásquez, Katia Zúñiga, Haydee Alvarado, Renso López-Liñán, Heriberto Arévalo, Nancy Rojas-Serrano

**Affiliations:** 1 Unidad de Innovación y Soporte Diagnóstico. Instituto Nacional de Salud, Lima, Perú.; 2 Facultad de Medicina Humana. Universidad de San Martín de Porres, Lima, Perú.; 3 Facultad de Ciencias Naturales y Matemáticas. Universidad Nacional Federico Villarreal, Lima, Perú.; 4 Laboratorio de Referencia Nacional de Virus de Transmisión Sexual. Instituto Nacional de Salud, Lima, Perú.; 5 Laboratorio de Referencia Nacional de Metaxénicas y Zoonosis Virales. Instituto Nacional de Salud, Lima, Perú.; 6 Hospital María Auxiliadora, Lima, Perú.; 7 Hospital Regional de Loreto, Loreto, Perú.; 8 Universidad Nacional de la Amazonia Peruana. Loreto, Perú.; 9 Laboratorio de Referencia Regional de Salud Pública de San Martín, San Martín, Perú.; 10 Laboratorio de Referencia Nacional de Enfermedades Inmunoprevenibles, Instituto Nacional de Salud, Lima, Perú.

**Keywords:** Coinfección viral, Infección por SARS-CoV-2, Síndrome Respiratorio Agudo Severo, Diagnóstico de Laboratorio

## Abstract

El estudio tuvo como objetivo identificar las coinfecciones de SARS-CoV-2 con otros virus en pacientes con COVID-19 moderada y grave. Se seleccionaron 98 pacientes de Lima, San Martín y Loreto, a quienes se tomaron muestras de secreción nasofaríngea, sangre y orina analizadas mediante pruebas serológicas y moleculares para detectar HTLV, VIH, hepatitis B, dengue, Chikungunya, Zika y un panel de virus respiratorios (influenza A/B, rinovirus, virus respiratorio sincitial, metaneumovirus y adenovirus). Los resultados mostraron dos casos de coinfección (2/98; 2%): uno con SARS-CoV-2/rinovirus y otro con SARS-CoV-2/HTLV-2. Ambos pacientes presentaron neumonía asociada a COVID-19, pero evolucionaron favorablemente. Se concluye que la frecuencia de coinfección viral en pacientes de establecimientos de salud de la costa y selva peruana con cuadros moderados y graves de COVID-19 fue baja.

## INTRODUCCIÓN

Después de su primer reporte, la COVID-19 continúa manifestándose en todo el mundo, con más de 778 millones de casos confirmados y 7,1 millones de fallecidos hacia finales de 2025 ^1^.

Durante 2020 y 2021, el 28,5 % de las muertes registradas a nivel mundial se concentraron en Sudamérica ^2^, siendo Perú el país con la mayor tasa de mortalidad per cápita por COVID-19 en el mundo ^3^.

Si bien la gravedad de la COVID-19 se ha asociado con factores metabólicos, genéticos y demográficos ^4^^-^^6^, recientemente se ha demostrado que las coinfecciones con otros virus respiratorios también contribuyen a una mayor severidad de la enfermedad ^7^^,^^8^. De hecho, se ha reportado que la tasa de letalidad en pacientes coinfectados con más de un virus fue tres veces mayor en comparación con aquellos que presentaron una sola infección ^8^. Estas evidencias sugieren que las coinfecciones virales pueden modificar el curso clínico de la enfermedad y constituirse en indicadores de mal pronóstico.

Cabe destacar que, durante la pandemia por SARS-CoV-2, Perú también afrontó diversas epidemias virales locales, entre ellas dengue (VDEN) ^9^, virus Chikungunya (VCHIK) ^10^, virus de la hepatitis B (VHB) y virus de la inmunodeficiencia humana tipo 1 (VIH-1) ^11^, virus linfotrópico de células T humanas (HTLV, por sus siglas en inglés) ^12^, así como virus respiratorios estacionales ^13^.

La circulación sostenida de estos agentes virales podría haber generado condiciones propicias para la ocurrencia de coinfecciones con SARS-CoV-2. En este contexto, con el objetivo de determinar la frecuencia de coinfecciones virales, se planteó un estudio multicéntrico en zonas donde también se han reportado otros virus endémicos, incluyendo sujetos con manifestaciones clínicas moderadas y graves de COVID-19.

**MENSAJE CLAVE**

Motivación para realizar el estudio. Durante la pandemia por SARS-CoV-2, la población peruana estuvo expuesta a diferentes agentes virales locales, incrementando la posibilidad de adquirir coinfecciones y, en consecuencia, riesgo de desarrollar la forma severa de COVID-19.

Principales hallazgos. Se reportaron dos casos de coinfección viral con rinovirus y HTLV-2. Este último, es el primer caso reportado en el mundo.

Implicancias. El presente estudio aporta con información sobre la presencia de agentes infecciosos virales que se presentan de manera simultánea en un contexto de pandemia y bajo características territoriales, lo cual es relevante desde un punto de vista clínico y epidemiológico.

## EL ESTUDIO

Se realizó un estudio transversal para reclutar pacientes con diagnóstico confirmado de SARS-CoV-2 mediante reacción en cadena de la polimerasa con transcripción inversa (qRT-PCR), entre el 26 de enero y el 13 de agosto de 2021. Los criterios de inclusión fueron edad ≥18 años, ambos sexos, hospitalización con COVID-19 moderada o grave según la Alerta Epidemiológica AE-016-2020 del MINSA. Se excluyeron pacientes con más de dos comorbilidades (diabetes, obesidad, hipertensión, cáncer).

Los pacientes fueron reclutados por conveniencia a partir de cuatro hospitales públicos: Hospital María Auxiliadora y Hospital San Juan de Lurigancho (Lima), Hospital II-2 de Tarapoto (San Martín) y Hospital Felipe Arriola Iglesias (Loreto). La selección de centros se realizó por conveniencia luego de una convocatoria nacional.

Las muestras incluyeron secreción nasofaríngea (para pruebas moleculares), sangre (5 mL en tubos sin anticoagulante) y orina (10-20 mL en frascos estériles). Todas fueron trasladadas en cadena de frío (0-10 °C) al Instituto Nacional de Salud en Lima para su procesamiento.

La recolección de datos se efectuó mediante fichas clínico-epidemiológicas COVID-19 aprobadas por el MINSA y el CIEI-INS.

Para el diagnóstico molecular de virus respiratorios se extrajo ARN de hisopados nasales y faríngeos con el kit QIAamp® DSP Viral RNA. Se aplicó qRT-PCR multiplex con sistema TaqMan en tres paneles: Panel 1 (Influenza A, Influenza B, GAPDH), Panel 2 (virus respiratorio sincitial, metaneumovirus, rinovirus) y Panel 3 (adenovirus). Las amplificaciones se realizaron en un termociclador RotorGene Q.

La detección de dengue (VDEN), Chikungunya (VCHIK) y Zika (VZIK) se efectuó mediante extracción de ARN viral en sueros con sistema automatizado de perlas magnéticas y posterior qRT-PCR con el kit Logix Smart™ ZDC. Para VZIK en orina se empleó el sistema Hollycon® AE2130 y qRT-PCR con cebadores dirigidos al gen E, utilizando termociclador BIORAD CFX96.

Las pruebas serológicas incluyeron detección de antígenos y anticuerpos contra VIH, HTLV-1/2, hepatitis B y arbovirus. Para VHB se usó ELISA HBsAg (WANTAI), para HTLV la prueba de Inmunoblot HTLV 1/2 (Fujirebio), y para VIH el kit Murex con confirmación mediante INNO-LIA™ HIV I/II Score. El diagnóstico serológico de dengue se realizó con ELISA IgM (TARIKI), mientras que para Chikungunya se empleó la prueba de inmunofluorescencia indirecta EUROIMMUN Chik IgM.

Este diseño permitió evaluar coinfecciones virales en pacientes hospitalizados con COVID-19 moderada y grave en diferentes regiones del Perú, bajo estrictos criterios éticos y metodológicos.

El estudio contó con aprobación ética del CIEI-INS (código OC-022-20). La participación fue voluntaria, con consentimiento informado firmado por pacientes o familiares, garantizando principios de ética médica e investigación científica.

## HALLAZGOS

Durante el periodo de reclutamiento se incluyeron 98 pacientes con diagnóstico confirmado de SARS-CoV-2, con estado clínico moderado o severo. La mayoría fue atendida en Lima (52%), aunque no se determinó si provenían de otras regiones. Predominó el sexo masculino (58,2%) y una edad promedio de 52 años. En cuanto a la presentación clínica, el 43,8% de los casos presentaron síntomas clínicos severos. Se identificaron comorbilidades en el 33% de los casos, principalmente diabetes (13%). Solo el 20,4% presentó pronóstico no favorable. La mayoría recibió tratamiento con enoxaparina y dexametasona (tabla 1).

**Tabla 1:** Características de los pacientes con COVID-19.

Características		HMA Lima n=27 (%)	HSJL n=24 (%)	Hospital II-2 de Tarapoto (San Martín) n=18 (%)	Hospital Felipe Arriola Iglesias (Loreto) n=29 (%)	Total n=98 (%)
Edad media (años)		59	48	52	49	52
Sexo						
	Masculino	17 (63,0)	15 (62,5)	8 (44,4)	17 (58,6)	57 (58,2)
	Femenino	10 (37,0)	9 (37,5)	10 (55,6)	12 (41,4)	41 (41,8)
Ventilación mecánica						
	Sí	0 (0,0)	1 (4,2)	2 (11,1)	1 (3,4)	4 (4,1)
	No	13 (48,1)	23 (95,8)	16 (88,9)	28 (96,6)	80 (81,6)
	ND	14 (51,9)	0 (0,0)	0 (0,0)	0 (0,0)	14 (14,3)
Pronóstico						
	Estable	11 (40,7)	5 (20,8)	9 (50,0)	6 (20,7)	31 (31,6)
	No favorable	5 (18,5)	4 (16,7)	4 (22,2)	7 (24,1)	20 (20,4)
	Favorable	9 (33,3)	13 (54,2)	5 (27,8)	14 (48,3)	41 (41,8)
	ND	2 (7,4)	2 (8,3)	0 (0,0)	2 (6,9)	6 (6,1)

HMA: Hospital María Auxiliadora; HSJL: Hospital San Juan de Lurigancho; ND: No determinado

Respecto a la coinfección viral, se detectaron dos casos (2%) (tabla 2). El primero correspondió a un hombre de 46 años en Lima con coinfección SARS-CoV-2/Rinovirus, cuadro moderado de neumonía y sin comorbilidades. Fue hospitalizado siete días y tratado con ceftriaxona, clindamicina y otros fármacos, evolucionando favorablemente. El segundo caso fue una mujer de 57 años en Iquitos con coinfección SARS-CoV-2/HTLV-2, que presentó tos, disnea e hiporexia. Sus signos vitales mostraron presión arterial 110/80, frecuencia cardíaca 107, respiratoria 26 y saturación 93%. Tras siete días de síntomas, recibió ceftriaxona, enoxaparina y omeprazol, con evolución positiva.

**Tabla 2:** Características clínicas y de laboratorio en los dos casos de coinfección viral.

Características	Primer caso	Segundo caso
Coinfección	Rinovirus	HTLV-2
Edad (años)	46	57
Clasificación clínica	Moderado	Grave
Comorbilidad	Ausente	Obesidad e hipertensión
Ventilación Mecánica	No	No
Evolución	Favorable	Favorable
Terapia farmacológica	Ceftriaxona, Clindamicina, Ketoprofeno, Tramadol, Ranitidina	Ceftriaxona, enoxaparina, azitromicina, dexametasona, metamizol y omeprazol
UREA (mg/dL)	ND	34
Creatinina (mg/dL)	ND	0,61
Glucosa (mg/dL)	ND	127,2
GOT (Ul/L)	ND	69
LDH (Ul/L)	ND	950
GPT (Ul/L)	ND	95
Proteína C reactiva (mg/L)	ND	134,8

ND, No determinado; GOT: Glutámico oxaloacetico transaminasa; LDH: Lactata deshidrogenasa; GPT, Glutamato-piruvato transaminasa.

## DISCUSIÓN

En este estudio realizamos una búsqueda de casos de coinfección viral en 98 pacientes hospitalizados positivos a SARS-CoV-2 a través de un estudio realizado en una ciudad de la costa y dos de la selva del Perú. Nuestros hallazgos revelaron la presencia de dos casos de coinfección viral (2%), cuya frecuencia está dentro del rango identificado por otros estudios (0,6 - 20%) ^7^^-^^9^^,^^12^^,^^15^^-^^18^. Sin embargo, la amplia diferencia de la frecuencia de coinfección viral puede deberse al número y tipo de muestras biológicas, las características de la población, el diseño del estudio, la presencia de infecciones virales endémicas e incluso los métodos diagnósticos empleados.

En el caso de García-Vidal *et al*. ^14^ encontraron una prevalencia de coinfección viral de 0,6% similar a nuestros hallazgos. Sin embargo, esto podría estar relacionado con el tipo de población seleccionada, ya que, en comparación con este estudio, nuestro reporte solo incluyó pacientes hospitalizados, en quienes también podría ocurrir coinfección bacteriana tal como se demostró previamente ^15^. Además, es probable que la detección de agentes virales esté sujeta a las limitaciones de la metodología utilizada, como en el caso del PCR en tiempo real cuya sensibilidad depende de la capacidad del emparejamiento de la secuencia de los *primers* y sondas en el ARN viral.

En cuanto a los casos identificados, la detección de coinfección entre SARS-CoV-2 con virus respiratorios, principalmente rinovirus, es relativamente frecuente ^16^. Aunque la infección por rinovirus no genera síntomas mayores en adultos, el caso de coinfección con SARS-CoV-2 exhibió una manifestación severa, lo cual es interesante porque el paciente no mostró comorbilidades. Al respecto, Le Glass *et al*.^16^ demostraron que los pacientes con coinfección por SARS-CoV-2 y rinovirus manifestaron formas clínicas severas, con mayor probabilidad de ingresar a cuidados intensivos y morir. Estos datos revelan que la coinfección por SARS-CoV-2/rinovirus virus podría desencadenar síntomas severos en pacientes adultos. Dado que el caso mostrado en nuestro estudio no presentó ningún tipo de comorbilidad y considerando la evidencia anterior, es posible que una coinfección entre SARS-CoV-2 y rinovirus haya sido la causa de la manifestación clínica grave en el paciente, sin embargo, esto requiere de mayores estudios.

Respecto al segundo caso, existen reportes previos de coinfección entre SARS-CoV-2 y HTLV ^12^^,^^17^^,^^18^, de los cuales Avila Dextre *et al.*^12^ reportó una posible coinfección entre el virus pandémico y HTLV-2. De acuerdo con ello, de un total de 22 portadores serológicamente confirmados por HTLV y SARS-CoV-2, siete muestras fueron tipificadas como HTLV-2. Sin embargo, la prueba de confirmación que utilizaron para diagnosticar la coinfección se basó en la detección de anticuerpos, por lo que no fue posible conocer con certeza el estado de infección por HTLV del paciente al momento de contraer SARS-CoV-2, principalmente por dos razones: 1) Desde el año 2000, más de 200 familias de comunidades nativas Shipibo-Konibo de la selva viven en Lima ^19^, una ciudad costera donde nunca se ha reportado HTLV-2. Esto sugiere, que no es posible saber si algunos individuos sanos en estas comunidades se infectaron con HTLV-2 antes o después de haberse infectado con SARS-CoV-2 o si algunos sujetos infectados con HTLV-2 estaban transmitiendo a otros de manera simultánea. 2) Los investigadores mencionaron que algunos casos positivos de SARS-CoV-2 fueron confirmados por prueba serológica IgM/IgG (ensayo ECLIA); sin embargo, estos pacientes fueron vacunados previamente contra el SARS-CoV-2, lo que aumenta la posibilidad de resultados falsos positivos. Todos estos argumentos apoyan el hecho de que nuestro estudio sería el primer informe confirmado de coinfección entre SARS-CoV-2 y HTLV-2 no solo en el Perú, sino también en el mundo. Sin embargo, tampoco tenemos la certeza de que se tratara de una coinfección aguda activa con SARS-CoV-2.

Analizando las características clínicas de este caso en particular, es importante señalar que el paciente coinfectado con SARS-CoV-2/HTLV-2 presentó una forma clínica grave de COVID-19, con una evolución favorable, el paciente presentó obesidad e hipertensión, factores que agravan la COVID-19 y por lo tanto podrían haber desempeñado un rol importante en las manifestaciones clínicas. En el reporte de Avila Dextre *et al.*^12^ siete sujetos presentaron manifestaciones clínicas moderadas; sin embargo, ninguno se asoció con la gravedad de COVID-19. Al respecto, el sesgo de memoria podría estar interfiriendo en la interpretación de estos resultados ya que se observó que la información clínica recolectada se basó en el recuerdo del participante. Por lo tanto, la evidencia es insuficiente para determinar si la presencia de HTLV-2 en un paciente con SARS-CoV-2 podría desencadenar formas clínicas moderadas o graves de COVID-19.

Es importante señalar que el presente estudio presenta algunas limitaciones. En primer lugar, el tamaño de muestra fue pequeño, principalmente por la dificultad de acceso a los pacientes en estado crítico, la negativa de los familiares y las restricciones de ingreso a la Unidad de Cuidados Intensivos durante la pandemia. En segundo lugar, la distribución heterogénea de los agentes virales a nivel nacional pudo haber limitado la detección de virus responsables de epidemias locales como DENV, CHIKV, ZIKV, VIH y VHB, cuyas manifestaciones clínicas pueden confundirse o exacerbar la COVID-19. En tercer lugar, la información clínico-epidemiológica de los sujetos seleccionados fue incompleta, debido al acceso restringido a la documentación clínica, la cual fue registrada por el personal asistencial de los establecimientos de salud como parte de la atención rutinaria, sin que estos formaran parte del equipo de investigación.

En conclusión, se encontró una baja frecuencia de coinfección viral en pacientes peruanos con formas moderadas y graves de COVID-19 en algunos establecimientos de salud de la costa y selva peruana. Estos hallazgos contribuyen con datos adicionales para el diseño de futuras estrategias de vigilancia epidemiológica, clínica y de laboratorio, que permitan identificar casos de coinfección viral. Asimismo, abre la posibilidad de nuevas líneas de investigación en el contexto de las coinfecciones y el desarrollo de estudios multicéntricos que fortalezcan la comprensión y respuesta frente a estos desafíos en salud pública.
